# Supplementary material for: Geodesics to characterize the phylogenetic landscape
Source: PLoS One. 2023 Jun 23;18(6):e0287350. doi: 10.1371/journal.pone.0287350 (PMC10289362; doi:10.1371/journal.pone.0287350)
Supplement: S1 File — (PDF) [file pone.0287350.s001.pdf]

Supporting Information for:

Geodesics to Characterize the Phylogenetic Landscape

Contents

1 Primate Dataset $\boldsymbol{D_1}$ . . . 2

1.1 Three trees chosen to illustrate pathtrees . . . 2

1.2 Best trees found by PATHTREES, PAUP*, and REVBAYES . . . 2

2 Snake Dataset $\boldsymbol{D_2}$: Best Trees Found by PATHTREES, PAUP*, and RAxML . . . 2

3 Effect of Interpolation Methods on Visualization . . . 4

4 Validating the MDS Visualization . . . 6

4.1 Validating for the primate dataset $\boldsymbol{D_1}$ . . . 6

4.2 Validating for the snake dataset $\boldsymbol{D_2}$ . . . 7

5 REVBAYES Script to Generate a Chain of Trees . . . 8

1 Primate Dataset D_1

1.1 Three trees chosen to illustrate pathtrees

Fig S1 shows the three trees that we have selected in Fig 3 to give an example of pathtrees.

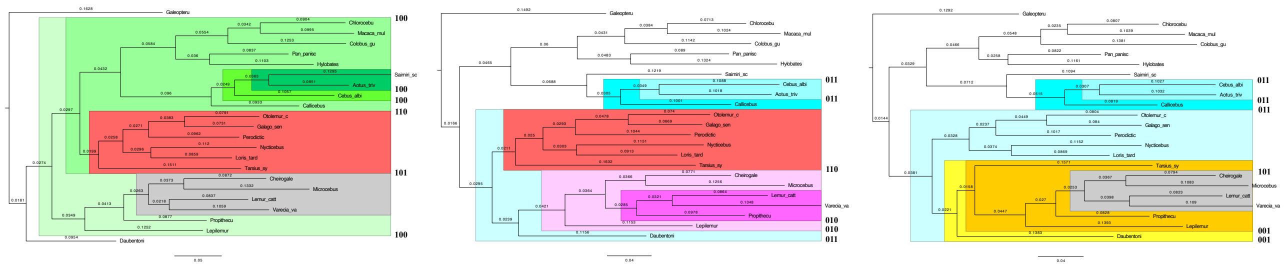

(a) tree ▲ (b) tree ▲ (c) tree ▲

Fig S1. The three trees used in Fig 3. Colors explain the similarities and differences of the splits in trees. Blue spectrum (011) shows subtrees in trees (b) and (c) and not in tree (a). Red color (110) shows the subtree in trees (a) and (b) and not in the tree (c). Grey color (101) shows the subtree in trees (a) and (c) and not in the tree (b). Green (100), pink (010), and yellow (001) spectrums show the subtrees just in tree (a), tree (b), and tree (c), respectively.

1.2 Best trees found by Pathtrees, Paup*, and RevBayes

For the primate dataset D_1, we compared our tree with the maximum posterior tree (MAP) of RevBayes and the best tree found by Paup*. As shown in Fig S2, our best tree and Paup* tree are the same, whereas MAP differs from both by two splits. These trees are shown on the likelihood landscape in Fig 5 and Fig 6.

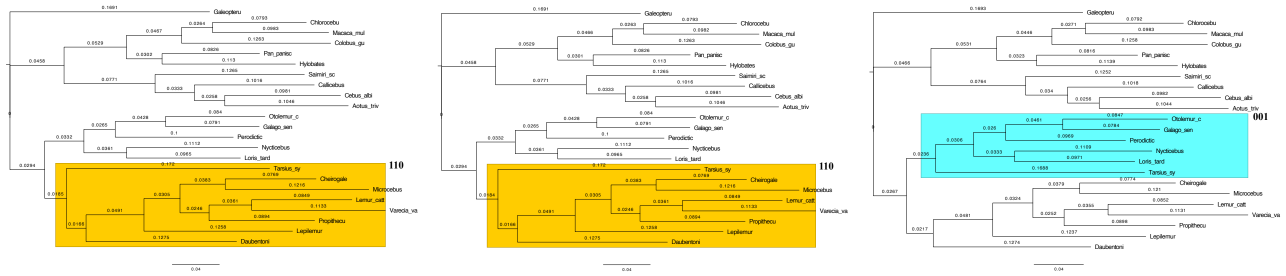

(a) Best tree by Pathtrees (b) Best tree by Paup* (c) Best tree by RevBayes

Fig S2. Best trees observed by Pathtrees (a), Paup* (b), and RevBayes (c) for dataset D_1. Yellow color (110) shows subtrees in the best trees found by Pathtrees and Paup* and not in the best tree of RevBayes. Blue color (001) shows the subtrees just in the best tree of RevBayes.

2 Snake Dataset D_2 : Best Trees Found by Pathtrees, Paup*, and RAxML

For the snake dataset, we compared our best tree with the best trees found by Paup* and RAxML. Fig S3 shows these three trees with colors showing the similarities and differences of the splits in trees.

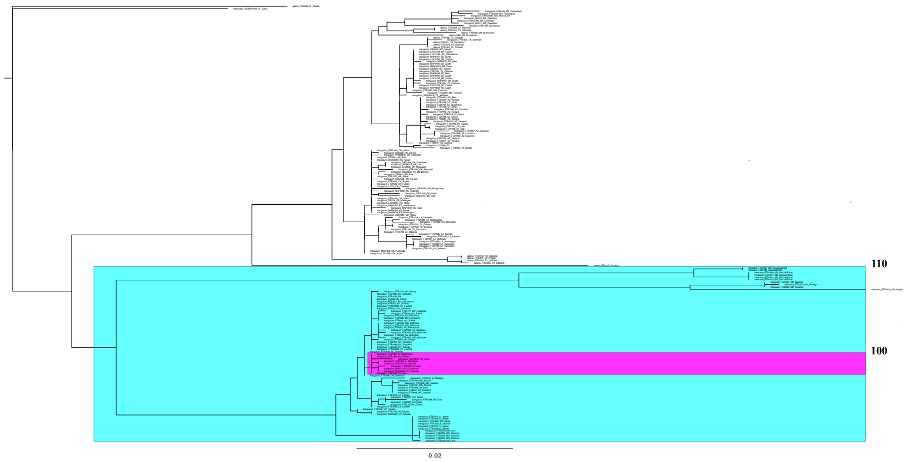

(a) Best tree by Pathtrees

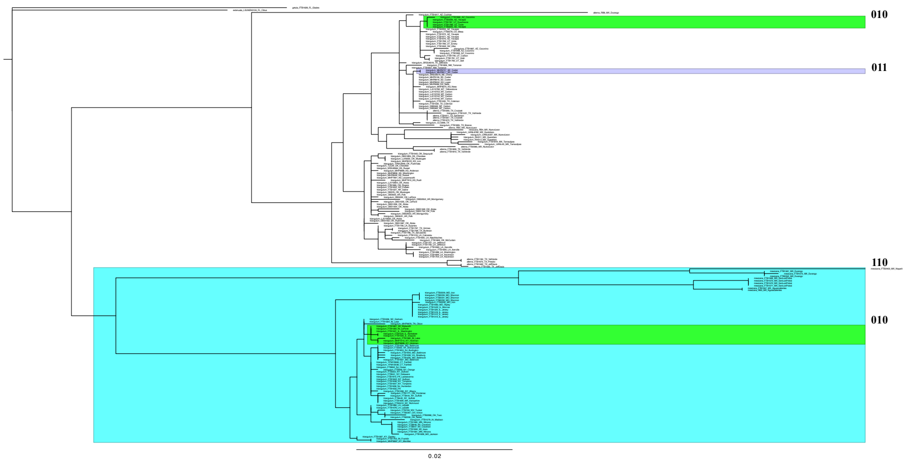

(b) Best tree by Paup*

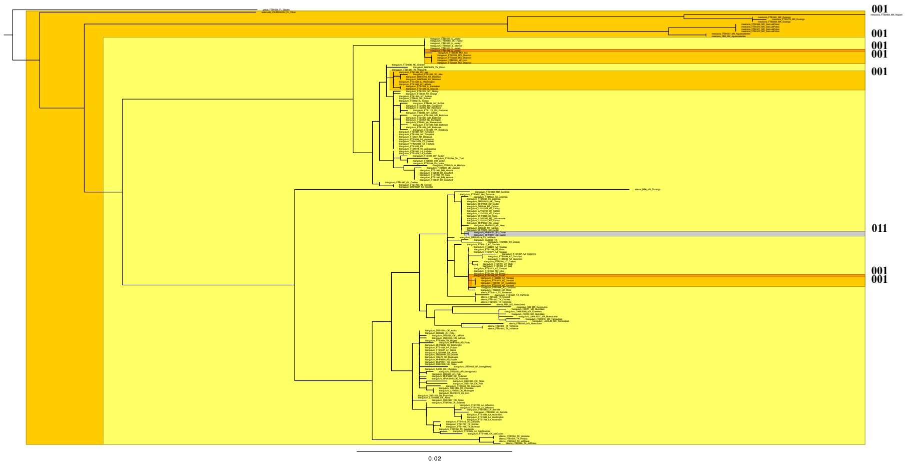

(c) Best tree by RAxML

Fig S3. Best trees observed by Pathtrees (a), Paup* (b), and RAxML (c) for dataset D_2. Blue color (110) shows the subtree in the best trees found by Pathtrees and Paup* and not in the best tree of RAxML. Purple color (011) shows the subtree in the best trees found by Paup* and RAxML and not in the best tree of Pathtrees. Pink color (100), green color (010), and yellow spectrum (001) show the subtrees just in the best tree of Pathtrees, Paup*, and RAxML, respectively.

3 Effect of Interpolation Methods on Visualization

Our package PATHTREES uses two different interpolation methods for the likelihood contour and surface. The default interpolation method is the RBF thin-plate spline with smoothness parameter of $s = 1e - 10$. The cubic spline interpolation can be used by setting the option `-interpolate cubic`, and the RBF thin-plate spline with any value of smoothness s can be used by setting the option `-interpolate rbf,s`. The thin-plate spline delivers surfaces that are less noisy. For example, comparing Fig 7 (thin-plate spline) and Fig S4 (cubic spline), the overall impression of the contour and surface features are similar but the cubic spline interpolation on the top row shows more extreme peaks than the thin-plate spline interpolation, and the distribution of the range of observed log-likelihood values on the color bar shows this clearly. Therefore, the RBF thin-plate spline interpolation gives a better impression of the true likelihood surface.

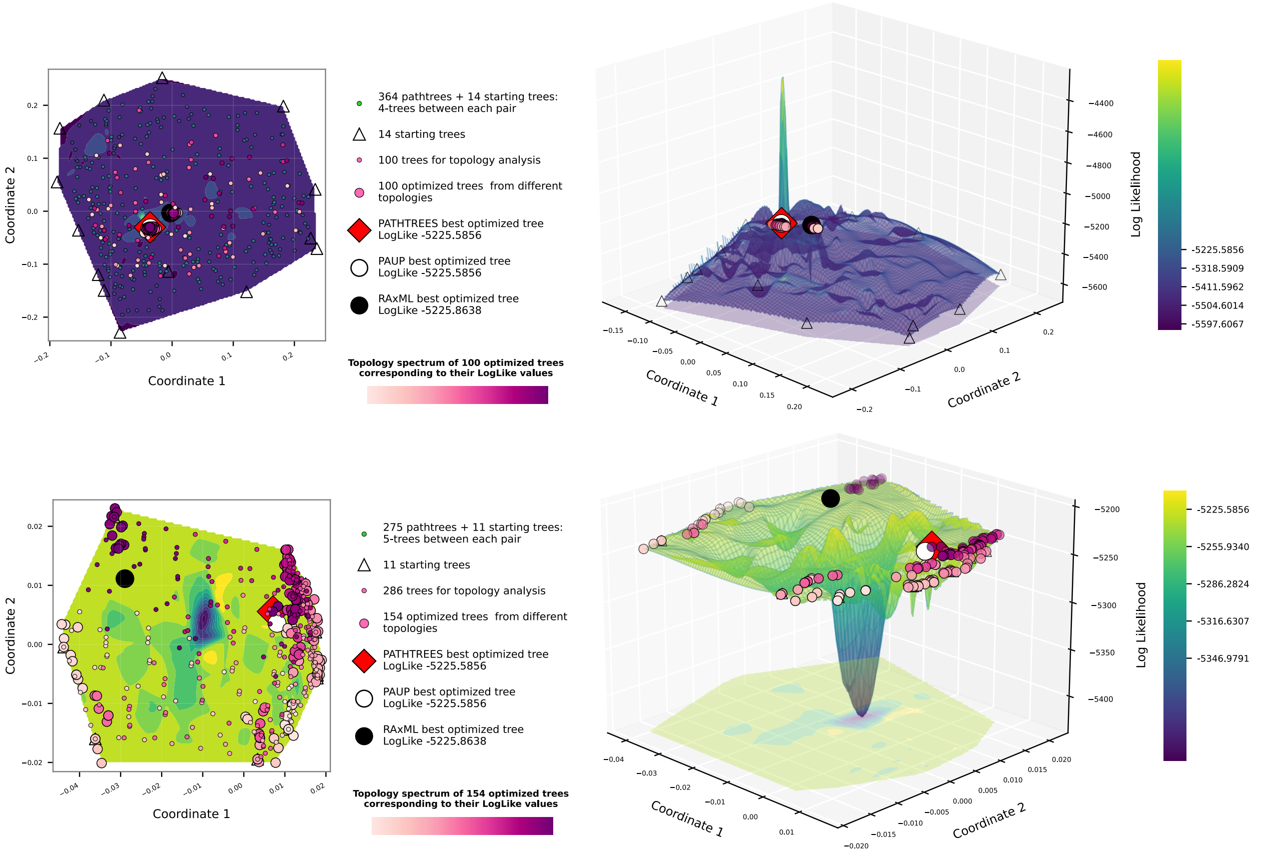

Fig S4. Contour and surface plots of PATHTREES for the dataset D_2 , using MDS and cubic spline interpolation defined by the weighted Robinson-Foulds distance metric. First row: the first iteration of PATHTREES using 14 starting trees. Second row: the second iteration of PATHTREES displaying the treespace after zooming inside the convex hull of 100 optimized trees from the first iteration.

Interpolation can fail if many trees are mapped close together in the MDS plot but have sufficiently different likelihoods. RBF will fail in such situations when the surface is forced through the observed data

points or will give a singular matrix error (Fig S5 bottom row). As a solution, increasing the smoothness parameter of the RBF interpolation from zero to a small value, such as 10^{-10} can lead to an acceptable visualization (Fig S5 middle row) compared to the cubic spline interpolation (Fig S5 top row). The cubic spline interpolation exaggerates the surface compared to the RBF thin-plate spline interpolation.

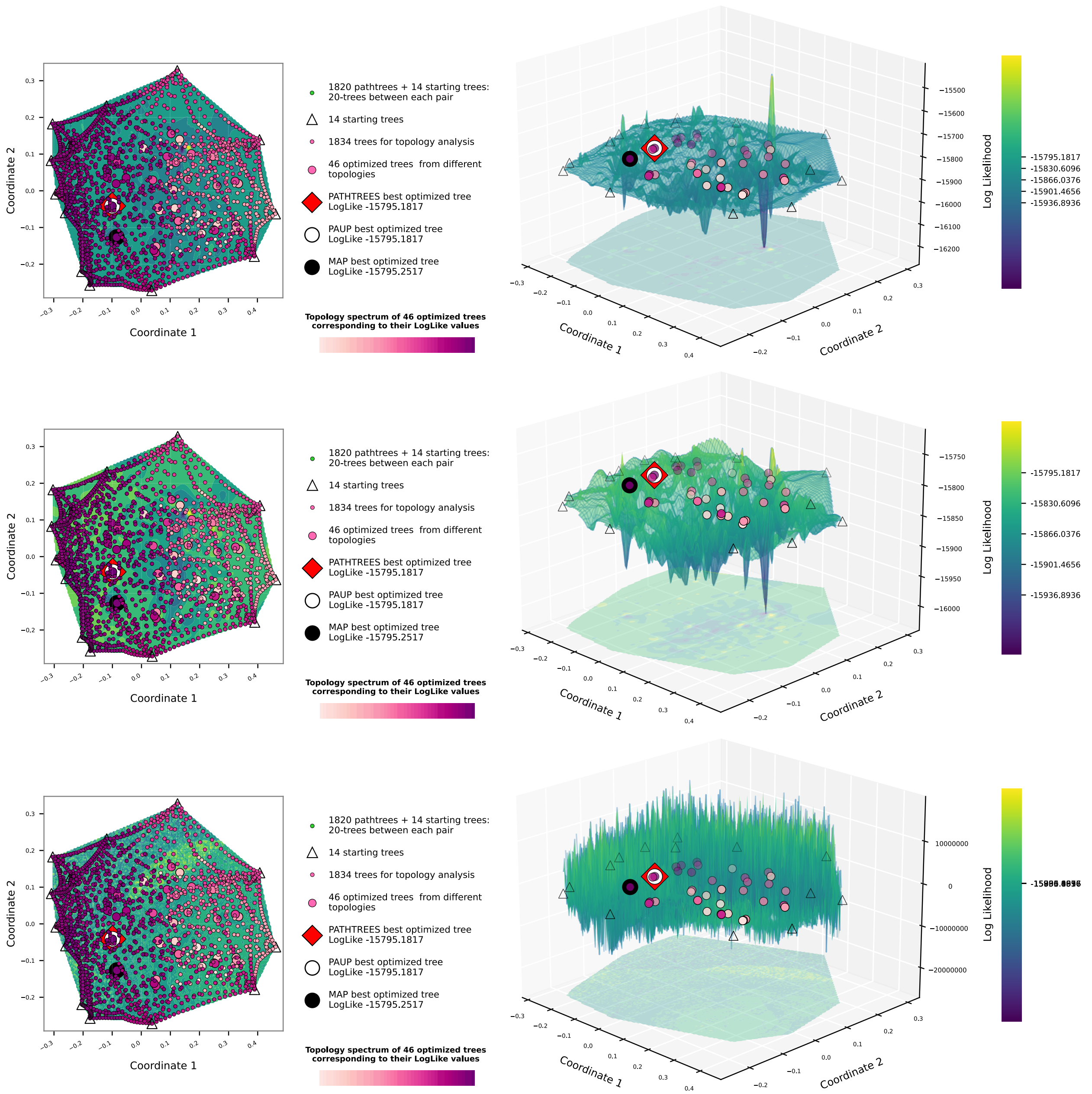

Fig S5. Contour and surface plots of Pathtrees for dataset D_1 by generating 20 pathtrees per anchor tree pair, using MDS, and (top row) cubic spline interpolation, (middle row) thin-plate spline with smoothness parameter of $s = 1e-10$, (bottom row) thin-plate spline with smoothness parameter of $s = 0.0$, defined by the weighted Robinson-Foulds distance metric.

We use MDS to place all trees onto a 2-D plane and then use the likelihood of the trees to interpolate the

likelihood tree landscape. This visualization of the MDS plane may differ dependent on the trees used and once in a while may deliver plots that are difficult to interpret; Fig S6 shows such a plot where the viewpoint of the plane shifted to be perpendicular to our original convex hull: we look at the convex hull sideways. In such cases, changing the number of optimized trees or the number of pathtrees between the anchor trees may help to remedy the view; for example, Fig 7A shows an adequate visualization by using fewer number of optimized trees compared to Fig S6.

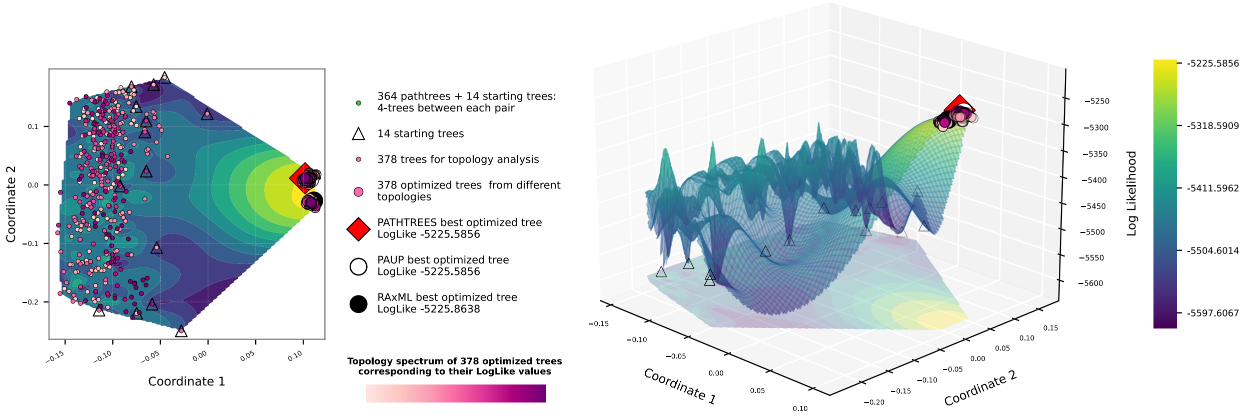

Fig S6. Contour and surface plots of PATHTREES for dataset D_2 , using MDS and thin-plate spline interpolation defined by the weighted Robinson-Foulds distance metric. Four trees were generated on the geodesic of each pair of 14 starting trees (364 pathtrees). All 364 + 14 trees were selected to be optimized. All trees have different topologies.

4 Validating the MDS Visualization

4.1 Validating for the primate dataset D_1

Fig S7 shows the Shepard diagrams of MDS distances vs. the original dissimilarities for Fig 2, Fig 3, and Fig 5B.

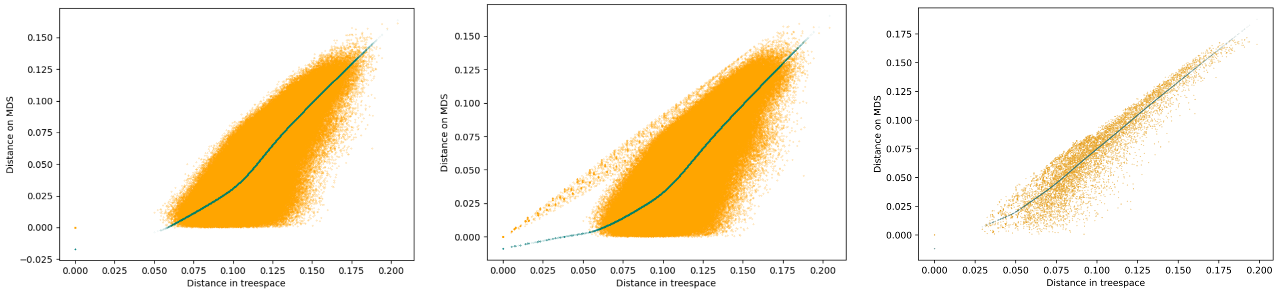

(a) (b) (c)

Fig S7. Shepard diagrams showing the BHV distances versus the MDS distances. (a) Shepard diagram of Fig 2. (b) Shepard diagram of Fig 3. (c) Shepard diagram of Fig 5B.

We computed the correlation measures Pearson r, Spearman rho, and Kendall tau between the original

distances and the MDS distances for Shepard diagrams in Fig S7.

For Fig 2:

Pearson's r $=$ 0.7869162205282654

Spearman's rho $=$ 0.7536010717168695

Kendall's tau $=$ 0.5643636289864661

For Fig 3:

Pearson's r $=$ 0.7713541497842118

Spearman's rho $=$ 0.7442553825613438

Kendall's tau $=$ 0.5533296366151566

For Fig 5B:

Pearson's r $=$ 0.9236859153718908

Spearman's rho $=$ 0.9071808059933395

Kendall's tau $=$ 0.7517609707944715

It can be seen that the correlation values of Pearson and Spearman are roughly the same, where the value of Kendall correlation coefficient is less than that of others. All these correlation methods are correct in terms of their values. Usually, Spearman's correlation value is closer to the Pearson's value than Kendall's value, and Kendall's value is less that the other two.

4.2 Validating for the snake dataset D_2

Here, for both iterations of Fig 7, the Shepard plots of MDS distances versus the real distances are shown in Fig S8 and the correlation measures Pearson r, Spearman rho, and Kendall tau are computed:

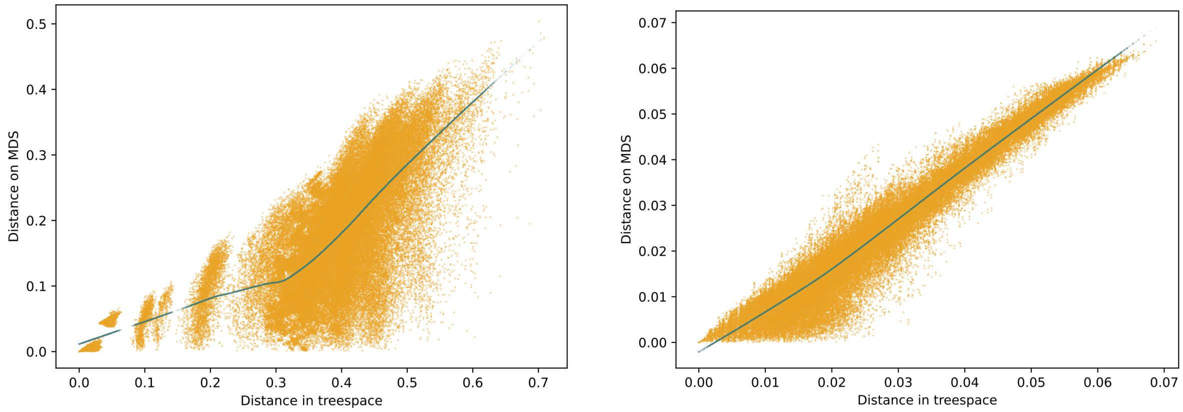

(a) Shepard diagram of the first iteration (b) Shepard diagram of the second iteration

Fig S8. Shepard diagrams of two iterations in Fig 7 , showing the BHV distances versus the MDS distances

First iteration:

$\text{Pearson's r} = 0.6855968829946086$

$\text{Spearman's rho} = 0.6954018788444843$

$\text{Kendall's tau} = 0.518946682776664$

Second iteration:

$\text{Pearson's r} = 0.9702223343002814$

$\text{Spearman's rho} = 0.9573847371609923$

$\text{Kendall's tau} = 0.832968379176889$

Since the figure iteration covers a much larger area of trees than the second iteration, the correlations measures are less than those in the second iteration.

5 RevBayes Script to Generate a Chain of Trees

This script is a shortened version of the tutorial page (Höhna et al. 2017)

```
################################################################################
#
# RevBayes Example: Bayesian inference of phylogeny using a Jukes-Cantor
#            substitution model on a single gene.
#
# authors: Sebastian Hoehna, Michael Landis, and Tracy A. Heath
# modified by Peter Beerli 2021
#
################################################################################


### Read in sequence data for both genes
data = readDiscreteCharacterData("data/primates_and_galeopterus_cytb.nex")

# Get some useful variables from the data. We need these later on.
n_species <- data.ntaxa()
n_branches <- 2 * n_species - 3
taxa <- data.taxa()



mvi = 1
```

```
mni = 1


########################
# Substitution Model #
########################

# create a constant variable for the rate matrix
Q <- fnJC(4)


###############
# Tree model #
###############

out_group = clade("Galeopterus_variegatus")
# Prior distribution on the tree topology
topology ~ dnUniformTopology(taxa, outgroup=out_group)
moves[mvi++] = mvNNI(topology, weight=5.0)
moves[mvi++] = mvSPR(topology, weight=1.0)

# Branch length prior
for (i in 1:n_branches) {
    bl[i] ~ dnExponential(10.0)
    moves[mvi++] = mvScale(bl[i])
}

TL := sum(bl)

psi := treeAssembly(topology, bl)



####################
# PhyloCTMC Model #
```

```
####################

# the sequence evolution model
seq ~ dnPhyloCTMC(tree=psi, Q=Q, type="DNA")

# attach the data
seq.clamp(data)



############
# Analysis #
############

mymodel = model(psi)

# add monitors
monitors[mni++] = mnScreen(TL, printgen=1000)
monitors[mni++] = mnFile(psi, filename="output/primates_cytb_JC.trees", printgen=10)
monitors[mni++] = mnModel(filename="output/primates_cytb_JC.log", printgen=10)

# run the analysis
mymcmc = mcmc(mymodel, moves, monitors)
#mymcmc.burnin(10000,200)
mymcmc.run(generations=500000)




####################
# Post processing #
####################

# Now, we will analyze the tree output.
# Let us start by reading in the tree trace
treetrace = readTreeTrace("output/primates_cytb_JC.trees", treetype="non-clock")
# and then get the MAP tree
```

```
map_tree = mapTree(treetrace,"output/primates_cytb_JC_MAP.tree")


# you may want to quit RevBayes now
q()
```
