# Supplementary material for: Geodesics to characterize the phylogenetic landscape
Source: PLoS One. 2023 Jun 23;18(6):e0287350. doi: 10.1371/journal.pone.0287350 (PMC10289362; doi:10.1371/journal.pone.0287350)
Supplement: Attachment — Submitted filename: Response_to_Reviewers.pdf [file pone.0287350.s002.pdf]

PLOS ONE: Response to Reviewers

Review Comments to the Author

Reviewer #1:

This is a very nicely written paper that was a pleasure to read. As far as I can tell the results are interesting, and novel, and likely to be of interest to those in the field of computational phylogenetic inference. **The comments below are all of a minor nature:**

- line 19-20. Finding trees not found with MCMC could be spurious - it's not obviously a good thing, though the authors make a strong case in the Discussion. maybe a few more words in the abstract, or even just use "relevant trees".

  We changed it to "relevant trees".

- line 51-52. The word "gappy" is obviously not technical and perhaps means something clear to the authors. It wasn't quite clear to me. Is this all with respect to BHV space? Likewise "some regions", refers to BHV space?

  We removed the word "gappy", made it more clear that "some regions" refer to regions in treespace (BHV or otherwise), and pointed the reader to Figure 2 as an example of uneven sampling. We also added text to emphasize that we don't seek to sample all of treespace (because it is much too large), but that previous work has found problems in sampling the relevant regions of treespace.

- line 89-90. This reads as though the sampling is across *all* geodesics, which is clearly not intended. Also "area of interest" is used a few times around here (line 89, 92, 115, 119, 121, 155, 173), and it wasn't clear at this point where this came from. Much later it is explained, but here it is opaque. Maybe somewhere explain that attention is focussed on an area of interest to explore, to motivate all this reference to it.

  We added an example of an "area of interest" where the term is first mentioned, namely "the region bounded by the trees from an MCMC run". We also added the word "certain" before "geodesics" to make it clear we were not taking all possible geodesics across the region.

- line 120. The "potentially" is doing a lot of work here. Sounds uncertain. Is there reason to hope?

  We changed the wording of this sentence to try to indicate that our algorithm can find trees of high likelihood unexplored by the tree search algorithms sometimes (as with dataset D2), but not others (as with dataset D1).

- line 193. Typo: "distance" matrix

  We fixed the typo.

- line 206. There is discussion about the closeness of trees in a 2D compression of tree-space. But how does this relate to actual tree-space? Trees close in 2D may be far apart in BHV space. But are trees far apart in 2D necessarily far apart in BHV? Does this compression have any nice isometric properties that mean it tells us something about the real space?

  For most of MDS visualizations, we compute the correlations between the tree distances and the MDS coordinate distances, and give these in the Supplementary File. These distances were generally highly correlated, suggesting the MDS visualizations are reasonable representations of the treespace. In Section

1.4, we added a pointer to the correlation in the Supplementary File for Fig 2 and Fig 3, as well as references to ongoing work to understand the relation between tree distance metrics and MDS visualizations.

- line 241. Where does the m come from? It's not an algorithm input.

  The parameter m is the number of trees on the geodesic between each pair of starting trees. Since it is an input given by user, we added it to the algorithm input section.

- line 244. Likewise where does n come from?

  The parameter n is the number of trees to select with the highest likelihoods among the current sample trees. Since it is an input given by user, we added it to the algorithm input section.

- line 267. Why 3/4? Earlier it was 1/10.

  We just wanted to give an example in Section 1.3, in Fig.2, that what is the process of extracting boundary trees (starting trees for PATHTREES) given some sample trees of chain (a random sample of 500-1000 trees from the chain is enough to extract boundary trees). Since we wanted to give a better picture of the area of best tree, we chose the last 1/10 of thee MCMC chain. You can choose another part of the chain. For example, as you find in Section 1.7, to show our results, we chose a different portion of chain (3/4) than Section 1.3. As you see, in Fig.2 we have 15 red dots and in Fig.5 we have 14 starting trees.

- line 273, 277. I don't understand why a smaller sample of 10,000 trees was taken when the data set has more taxa than D1. Also, for consistency, in line 277 use a comma in "10000".

  We revised the sentences and tried to make it more clear.

- line 296. Italics for a posteriori?

  We put "a posteriori" in Italics in the two places it appears in the paper.

- line 424. "lie" instead of "lay"?

  Fixed.

Reviewer #2:

Main Ideas: This work aims to provide a new visualization method for phylogenetic trees that allows for the construction of intermediate trees connecting two given trees. They do this by mapping treespace into lower dimensions means representing a high-dimensional space (space of all possible trees) in a lower-dimensional space (e.g., a 2D or 3D space) to make it easier to visualize and explore. The authors provide a new method for constructing intermediate trees between two trees in BHV space.
The proposed visualization method is effective and potentially has applications exceeding the scope of this work. This work focuses on applications to ML and Bayesian tree searches. The authors compare it with existing methods such as PAUP*, RAxML, and RevBayes. They use primate and milk snake datasets and show that their method can find trees that were not found with heuristic search methods.

General Feedback: The paper is well-written and informative from both the mathematical and biological perspectives. Given the readily available software, the results are new and likely to be used. It is a notable f interdisciplinary project combining data science, mathematics, and biology.

However, the most significant limitation of the current version of the article is that the audience needs to be made more explicit. Without framing the implications of this work, it is likely to be encountered by mathematicians who wish to know more about the geometry of the mapping from BHV space to R2, And by practitioners who may want to know the exact scenarios under which they should be using this tool and to have more evidence that it can be relied upon to be helpful. However, doing either of these, much less both, would be more work than is appropriate for a revision and would likely warrant separate publications. So instead, I recommend this paper be accepted with relatively minor revision per the suggestions below.

Concerns:

- Revise the framing of the introduction in a manner that clearly sets out what a reader can expect from the paper in terms of usability and mathematical nuance.

  We added a section towards the end of the introduction that points towards uses for biologists.

- There should be more discussion regarding potential use for biologists both now and in the future. The software is there, but it needs to be advertised more in the paper so people will use it.

  We have emphasized the software in several places.

- Line 357-363, This statement is troubling because one of the more significant results is that the pathtree method found an optimal tree that another method missed. However, it needs to be clarified if RAxML missed this more optimal tree intrinsically or missed it due to the switching between models.

  The search in RAxML *is not done under the same exact model as in* Paup*. *We constrained for the same mutation model JC69, but it seems that the search still happens under a site-rate variation constraint (GTRCAT), leading to different topologies. There is an extensive discussion in the raxml manual about GTRCAT and GTRGAMMA that we find marginal for our description of treespace maxima. All found 'best' trees are within 1 log-likelihood unit.*

Minor Concerns:

- The authors could provide a picture of the "gappy" space to help readers understand the problem. The term might also confuse someone who thinks it refers to the sequences having many gaps.

  We removed the word "gappy", made it more clear that "some regions" refer to regions in treespace (BHV or otherwise), and pointed the reader to Figure 2 as an example of uneven sampling. We also added text to emphasize that we don't seek to sample all of treespace (because it is much too large), but that previous work has found problems in sampling the relevant regions of treespace.

- The authors should clarify technical terms (such as topology proportions, consistency of generated treespace, and artifact). It sometimes took work to tell when these were being used formally or informally.

  We revised the abstract to be more clear, removing the phrases "topology proportions" and "consistency of generated treespace", since we do not use them in the paper. Due to the word count limitations for the abstract, we had to make some other minor changes. For "artifact", we revised the wording the first place it was used, as it was being used incorrectly. We added a brief definition after the second (now first) use.

- The authors should explain more about the clustering results and compare them with RF distance results.

  We revised the review of previous MDS visualization in the introduction to emphasize that the trees can be colored by qualitative or quantitative descriptors, and that this is independent of the choice of tree distance metric. We also fixed an incorrect reference.

- The authors could provide more details on the relationship between the BHV space and the MDS plane. In particular, there are choices of metrics in multiple stages of the Algorithm, and it needs to be clarified what the implications of the choices may be

  While this is an important point, it is also a hard question to answer and an area of ongoing research. We added a pointer to this research in Section 1.4. For most of MDS visualizations, we compute the correlations between the tree distances and the MDS coordinate distances, and give these in the Supplementary File. These distances were generally highly correlated, suggesting the MDS visualizations are reasonable representations of the treespace.

- Line 197: Get rid of "using an interpolation method" since that is clarified in the following sentence.

  The comment is applied.

- Line 185 is "not" an algorithm. Also, move this sentence above the example using REV Bayes. Using distance methods as an example of searching through tree space as an optimization problem seems like an alternative theoretical problem to that described here.

  We added the "not" and moved the sentence as suggested.

- Namely, in the ML and Bayesian Contexts, you search through trees and check a function's value. In contrast, in distance-based methods, one uses finite data to build a tree, typically in some sequential order, to (potentially) optimize something, thus avoiding even the intention of a full global search. Readers will likely understand this work more clearly in the context of the ML/Bayesian methods.

  We may have misinterpreted this comment. Our approach does not rely on a data distance metric but instead focuses on likelihood and the relationships among trees in BHV space. We are confident that readers of this manuscript will not encounter any confusion regarding the context.

- Section 1.5 Explain why we need path trees with the same topology. If I understand this section correctly, it looks like the optimized trees (same topology as the two consecutive path trees) can appear quite far away in this diagram.

  We were unclear, and have revised the beginning of Section 1.5 to say that we need to find all distinct pathtree topologies, and then optimize each different topology.

- Line 121: Points "on" a convex Hull. Does that mean points on the boundary of the convex hull, vertices, or any points in the convex hull?

  We corrected that to the vertices of a convex hull

- Line 263 Consolidate the dataset into unique site patterns. Does this mean not only using SNPS or not excluding individuals whose sequences match?

  We changed the wording of this sentence to "We deliberately did not remove individuals that have identical sequences".

- Line 307: Using "our method twice" in this sentence is not needed.

  The duplicate is removed.

- In the results section, it might be easier to refer to D_1 and D_2 as the primate and milk snake datasets.

  We revised them in the paper, and now we refer them to primate dataset D1 and snake dataset D2 in most of occurrences.

- Line 346: Should it be "The 11 trees"

  The comment is applied.

- In terms of your figures, you indicate that the path trees correspond in clusters to tree topologies. It would be interesting to see how tree topology differences map into the R2 space as an overlay in these examples or toy mapping. The reason is that there would be more interest biologically, in very close local maxima that correspond to differing topologies, than in local maxima corresponding to different branch lengths.

  We already color the trees selected in step 5 of our algorithm by their topology. Since this was not clear, we have explicitly stated that we color by topology in step 7 of the algorithm, and also revised how we describe the coloring in the introduction and beginning of the results section.

- In the correlation coefficient section, Page 16, it would be helpful to have a reference indicating that the Person coefficients or Shepard diagrams are standard measurements when reducing the data dimension.

  We added a sentence with two references to section 1.4, where we are referring to MDS validation for the first time.

- Paragraphs 373-380 would help interpret the results to know how many different topologies were among the REVBayes trees.

  For the primate dataset D_1, there are 168 different topologies in 50000 RevBayes trees. For the snake dataset D_2, all 10000 RevBayes trees are having different topologies. These results are added to the corresponding Paragraph.

- Line 383: I love the term "augment an ML tree search" Perhaps this term is the key to clearly framing the paper.

  We made many changes to the manuscript and hope that these changes help the readers. We believe that an extension of our work will improve current ML searches.

- Paragraph 403, I am not sure about the latest version of Astral, but earlier versions had the limitation of requiring the splits of the species tree to be among the set of splits of the gene trees. They showed that this is not an issue in practice or even theoretically. They do not need to redo their result; putting this in a familiar context for a user is helpful.

  Thank you for this pointer. We have added reference to it in to give further context.

- In this mapping, it may be helpful to pose some of the open questions regarding the relationship between BHV space and R^2.

  We added a pointer to ongoing work to understand the relation between tree distance metrics and MDS visualizations in Section 1.4.
